# Commentary on Chronic Kidney Disease and Increased LAVI as Risk Factors of New‐Onset Heart Failure in Atrial Fibrillation: A Case–Control Study

**DOI:** 10.1002/joa3.70142

**Published:** 2025-07-15

**Authors:** Hafsa Ali, Manayim Fatima, Muhammad Saeed Qazi, Tazeen Saeed Ali, Javed Iqbal

**Affiliations:** ^1^ Sindh Medical College Jinnah Sindh Medical University Karachi Pakistan; ^2^ Medical Research Center Liaquat University of Medical and Health Sciences Jamshoro Pakistan; ^3^ School of Nursing and Midwifery Aga Khan University Karachi Pakistan; ^4^ Nursing Department Hamad Medical Corporation Doha Qatar

**Keywords:** atrial fibrillation, chronic kidney disease, heart failure

To the Editor,

It was interesting to read the article Chronic Kidney Disease and Increased LAVI as Risk Factors of New‐Onset Heart Failure in Atrial Fibrillation: A Case–Control Study, by Resultanti Irwan Muin et al. [1] This study discusses a significant association between chronic kidney disease (CKD) and increased left atrial volume index (LAVI) with new‐onset heart failure (HF) in individuals with existing atrial fibrillation. Determining and detecting these risk factors is essential for the prevention and prognosis of HF in AF patients. However, several methodological aspects warrant further discussion.

First, the study adopts a retrospective case–control design based on a single institution, with several limitations. Retrospective data relies mainly on the patient's memory when interviewed and can often introduce a recall bias. The authors did not mention blinding in the study design, which could lead to an interview or measurement bias. As the study analyzes patients from a single institution, it is not population‐based; therefore, generalizing the results and computing incidence is impossible [2]. Moreover, selection bias is possible as the control group was picked randomly using a computerized generator (www.random.org), whereas the case group was selected using purposive sampling. Adding on, the study's small sample size, that is, 132 patients in total and only 44 cases, considering that there were 9110 records retrieved for AF, out of which 6465 AF patients have HF, limits the statistical power and generalizability of the study [1].

Secondly, the study reports significant differences in the AF type and duration between the case and control groups. Persistent AF was more prevalent (43.2%) in the case group, while paroxysmal AF was more common (50%) in the control group; these were not accounted for in multivariate modeling. Persistent AF and longer duration are individual risk factors for HF and are linked to a higher incidence of HF compared to paroxysmal AF [3]. Failing to adjust for them might lead to a complication when determining the association with CKD and LAVI.

Thirdly, while LAVI was used as a predictive variable, it was only measured once after the diagnosis of AF. The authors did not state the timing or consistency of this echocardiographic evaluation. LAVI might fluctuate based on body size, age, blood pressure, medical history, tobacco and alcohol use, diastolic dysfunction, and technician variability. The lack of standardization can compromise confidence in the utility of LAVI as a stable predictor [4]. We understand that retrospective data often limit measurement frequency, but acknowledging this in interpretation could improve balance. Moreover, medication data are absent; the authors have not mentioned the usage of any medications for AF and HF, which can significantly impact both HF and LAVI outcomes.

Finally, for statistical analysis, we appreciate the authors' use of basic statistical models such as logistic regression to determine which factors increased the risk of HF. However, complementary statistical techniques such as receiver operating characteristic ROC analysis may improve predictive assessment. The ROC curve can be implemented to show how well a factor, such as LAVI, predicts who has an increased risk of HF. The area under the curve (AUC) is an overall combined metric of the ROC curve that shows the accuracy of tests like LAVI in differentiating between HF and non‐HF individuals when selected randomly from the sample. AUC values of more than 0.9 indicate the excellent diagnostic capability of the tests [5]. A sensitivity analysis checks if the results are valid under different conditions. To make findings more robust, they must be insensitive to changes in methodology and analysis. Sensitivity analysis could give readers more confidence in the methods, analytics, and measures used by the researcher [6]. Although not essential, such tools can enhance the understanding of diagnostic accuracy.

In conclusion, while the study raises important questions on the renal and structural risk factors of HF in patients with AF, some limitations compromise the quality and generalizability of results, leading to a need for more rigorous, prospective, and statistically robust research. We commend the authors for addressing an understudied area and encourage continued investigation using standardized methods and multivariable risk modeling.

## Ethics Statement

The authors have nothing to report.

## Conflicts of Interest

The authors declare no conflicts of interest.
